# Toxic and Potentially Toxic Mineral Elements of Edible Gastropods Land Snails (Mediterranean Escargot)

**DOI:** 10.3390/toxics11040317

**Published:** 2023-03-28

**Authors:** Roberta Tardugno, Antonino Virga, Vincenzo Nava, Federica Mannino, Andrea Salvo, Francesco Monaco, Mario Giorgianni, Nicola Cicero

**Affiliations:** 1 Department of Pharmacy—Drug Sciences, University of Bari, 70121 Bari, Italy; 2 Department of Agricultural, Food and Forestry Sciences, University of Palermo, 90121 Palermo, Italy; 3 Department of Biomedical, Dental, Morphological and Functional Image Sciences (BIOMORF), University of Messina, 98100 Messina, Italy; 4 Department of Clinical and Experimental Medicine, University of Messina, Via C. Valeria, 98125 Messina, Italy; 5 Department of Chemistry and Drug Technology, University of Roma La Sapienza, 00185 Roma, Italy; 6 Science4life srl, University of Messina, 98100 Messina, Italy

**Keywords:** gastropods, snails, minerals, chemical analysis, ICP-MS, DMA-80

## Abstract

The meat of snails can be considered a high-quality food for the human diet and demand is already increasing across Europe. Due to the bioaccumulation of trace elements in their tissues, land snails can be a significant tool also for environmental pollution evaluation. In this study, 28 mineral elements (Ag, Al, As, B, Ba, Be, Bi, Cd, Co, Cr, Cu, Fe, Hg, K, Li, Na, Mg, Mn, Mo, Ni, Pb, Sb, Se, Sr, Ti, Tl, V, Zn) in both the edible part and the shell of edible land snails commercially available in Southern Italy belonging to *Cernuella virgata*, *Helix aperta*, *Theba pisana* species were investigated by ICP-MS and direct mercury analyser. The concentration of trace elements was variable among the samples. The variability demonstrates the close connection among the type of snail, the geographical origin, and the habitat in which the species grows. The edible part of the snails analysed in this study was found to be a good source of macro-nutrients. Toxic elements were detected in some samples, especially in shells; nevertheless, the values fell within the safety limits. Further investigations and monitoring of mineral contents in edible land snails are suggested both for human health and environmental pollution evaluation.

## 1. Introduction

Snails are invertebrate animals present in human diet, culinary habits, and medical preparation since prehistoric times worldwide [1,2]. Aquatic, marine, and terrestrial edible snails belong to the class *Gastropoda* and phylum *Mollusca*. About 80,000 species of gastropods are known, with varied body structures and sizes. The body is often soft, with a calcareous shell for defensive purposes [3]. In European countries, land snails are part of traditional cuisine in Greece, France, and Italy. They are principally prepared by roasting and boiling according to local recipes [4]. The most consumed land snails belong to the genera of *Helicidae*, such as *Helix Aperta*, *Theba pisana* [5], and *Geomitridae*, such as *Cernuella virgata*. *Theba pisana* is a widespread species throughout the Mediterranean basin. It is found in dune environments, but also near rivers. It possesses a robust, whitish shell with brown bands, with a diameter of 15–20 mm and a height of 10–15 mm. The natural habitat for *Helix aperta* is represented by Mediterranean scrubland. In fact, it is found in southern Europe and North Africa. It has a greenish/brown shell. *Cernuella virgata* is a very common species in northwestern Europe and the Mediterranean basin and usually lives in dry environments. It has a yellowish-white shell with one or more brown or blackish bands, 12–23 mm in diameter and 8–15 mm in height. Generally, the shells are a good source of calcium, and find many fields of application from animal feeding to cosmetic industries, as well as ornaments in restaurants [1,2]. Land snails can be principally found in markets, and in addition, the collection of wildland snails is considered a traditional activity during the rainy periods. Is estimated that the domestic European market cannot cover the entire demand for this niche food product [2,6]. Many tons of land snails are imported from African and Middle East countries. [4]. The demand for land snails has been gaining again in the last decades, thanks to the progress of gastronomy and modern snail farming [2,6]. Their nutritional value and sustainable characteristics are the features that attract cultivators, researchers, and consumers as an alternative source of fundamental nutrients [2,4].

The meat of snails can be considered a high-quality food for the human diet due to the presence of high amounts of protein and minerals, a relatively low lipid content and a balanced intake of omega-3 and omega-6 fatty acids [4,7]. The composition of snail meat may change under the influence of its living conditions. Among minerals, edible snails can accumulate higher concentrations of some beneficial micronutrients such as selenium (Se), copper (Cu) and zinc (Zn) for human nutrition. On the other hand, the snails can also bioaccumulate heavy metals in their tissues; in particular, contaminated soils and cooking processes seem to increase the content of toxic elements such as cadmium (Cd) and lead (Pb) in the snail meat, transforming the snail meat from a beneficial alternative ecological food to a toxicological risk [7,8,9]. Their mineral content is strongly related to several factors: not only to geographic origin and diet, but also to seasonal and biological cycles. In addition, genetic and physiological variability factors among species influence the biochemical constitution of snail tissues and, consequently, their nutritional composition [5].

In addition, understanding terrestrial biota and its changes over time is a crucial part of the long-term carbon cycle through the deposition of biomass such as coal and other sedimentary substances, including mineral elements and the impact of plants, fungi and microbial life on silicate mineral erosion [10].

Edible snails can be considered sentinel species, due to the bioaccumulation of trace elements in snail tissues, also being a significant tool for environmental pollution evaluation and the health of terrestrial biota [7,9,10,11,12,13,14]. The high quantity of toxic minerals in the environment tends to significantly affect the mineral composition of land snails and makes them one of the living organisms most susceptible to possible bioaccumulation of toxic elements, which could create health problems for the consumer. Given the high rate of consumption of this matrix, it is therefore appropriate to know both its nutritional value and level of toxicity on humans [5].

In this context, the mineral content variability in land snails, due to high nutritional, environmental, and economic value as a niche food, play a key role.

The objective of this study was to determine 28 mineral elements (Ag, Al, As, B, Ba, Be, Bi, Cd, Co, Cr, Cu, Fe, Hg, K, Li, Na, Mg, Mn, Mo, Ni, Pb, Sb, Se, Sr, Ti, Tl, V, Zn) in both the edible part and the shell of edible land snails commercially available in Southern Italy belonging to *Cernuella virgata*, *Helix aperta*, *Theba pisana* species.

## 2. Materials and Methods

### 2.1. Samples

In this study, edible snail samples were purchased from different local markets in Messina and Palermo (Sicily, Palermo, Southern Italy). Specimens were identified based on morphological features. The snail samples belonged to three different species: *Theba pisana*, *Helix aperta* and *Cernuella virgata*. Table 1 shows the samples investigated, referring to their species, the area where they were purchased and their area of origin, when indicated. A total of 12 samples have been analysed: 2 *T. pisana*, 8 *H. aperta* and 2 *C. virgata* samples, respectively. For each sample, the edible part was separated from the shell. Ethical guidelines regarding the treatment of animal for experiments were strictly followed for the analysis. In fact, the fewest possible number of samples in compliance with the ethical guidelines was used for successful experimental analysis.

### 2.2. Materials and Reagents

Supra-pure-grade hydrogen peroxide (H_2_O_2_, 30% *v*/*v*) and nitric acid (HNO_3_, 65% *v*/*v*) were purchased from J.T. Baker (Mallinckrodt Baker, Milan, Italy), and hydrochloric acid (HCl, 37% *v*/*v*) and ultrapure water from Merck-Millipore (Darmstadt, Germany). The commercial standard solution of Re (internal standard), and the standards of Ag, Al, As, B, Ba, Be, Bi, Cd, Co, Cr, Cu, Fe, K, Li, Na, Mg, Mn, Mo, Ni, Pb, Sb, Se, Sr, Ti, Tl, V, Zn, used for the calibration curves, were purchased from Supelco (Bellefonte, PA, USA).

From Merck (Darmstadt, Germany) a Hg solution (1000 mg/L in 3% hydrochloric acid) was purchased. To clean the DMA-80 instrument, 3% HCl solution, prepared from concentrated HCl (37%), obtained from Merck (Darmstadt, Germany), was used.

### 2.3. Element Analysis

To determine the mineral element content, samples were initially subjected to a pre-treatment step based on an acid digestion process conducted in a closed-vessel microwave digestion system (ETHOS 1, Milestone, Bergamo, Italy). Mineralization procedures varied depending on the part of the snail being considered. Regarding the edible part, approximately 0.5 g of each sample was accurately weighed in PTFE vessels, fortified with 1 mL of internal standard Re at a concentration of 0.5 mg/L, and mixed with 7 mL of HNO_3_ and 1 mL of H_2_O_2_. The operating conditions of the digestion program were: 15 min at 0–180 °C (step 1), 15 min at constant 180 °C (step 2). Both steps had a microwave power of 1000 W. For the snail shells, 1 mL of Re internal standard at a concentration of 0.5 mg/L, 6 mL of HCl and 2 mL of HNO3 were added to approximately 0.5 g of each sample. The operating conditions were as follows: 10 min at a temperature varying between 0 and 180 °C (step 1), 10 min at a constant 200 °C. Again, the microwave power was constant at 1000 W. For each mentioned pre-treatment method, the third step consisted of a 20 min cooling phase.

Subsequently, samples were diluted to a volume of 25 mL with ultrapure water and filtered through 0.45 μm PTFE filters. Both the blank solution (HNO_3_ and H_2_O_2_ (7:1 *v*/*v*) for the edible part, HCl and HNO_3_ (6:2 *v*/*v*) for the shells) and the certified reference material (ERM-CE278k- Mussel Tissue, Merck Spa, Sigma-Aldrich, Milan, Italy) were prepared under the same conditions as the samples.

### 2.4. ICP-MS Analysis

The certified reference material (ERM-CE278k-Mussel Tissue) contained the following mineral elements: As, Cd, Cr, Co, Cu, Fe, Pb, Mg, Mn, Hg, Ni, K, Se, Ag, Na, Sr, Zn.

Ag, Al, As, B, Ba, Be, Bi, Cd, Co, Cr, Cu, Fe, K, Li, Na, Mg, Mn, Mo, Ni, Pb, Sb, Se, Sr, Ti, Tl, V, Zn were determined by the single quadrupole inductively coupled plasma-mass spectrometer (ICP-MS, iCAP-Q, Thermo Scientific, Waltham, MA, USA) powered by a 27 MHz radiofrequency solid-state generator, and equipped with a PFA cyclonic spray chamber with a port accepting a 4 mm i.d. and 6 mm o.d. nebulizer, nickel sampler and skimmer cones of 1.1 mm and 0.5 mm. The instrument was also provided with an autosampler (ASX520, Cetac Technologies Inc., Omaha, NE, USA) coupled to an integrated sample introduction system.

The following isotopes were monitored for ICP-MS analysis: ^7^ Li, ^9^ Be, ^11^ B, ^23^ Na, ^24^ Mg, ^27^ Al, ^39^ K, ^48^ Ti, ^51^ V, ^52^ Cr, ^55^ Mn, ^56^ Fe, ^59^ Co, ^60^ Ni, ^63^ Cu, ^66^ Zn, ^75^ As, ^80^ Se, ^88^ Sr, ^98^ Mo, ^107^ Ag, ^114^ Cd, ^121^ Sb, ^138^ Ba, ^205^ Tl, ^208^ Pb and ^209^ Bi.

Snail samples were analysed under the following operating conditions: RF power, 1550 W; plasma gas (Ar) flow rate, 14 L/min; auxiliary gas (Ar), flow rate, 0.8 L/min; carrier gas (Ar) flow rate, 1.1 L/min; collision gas (He) flow rate, 4.7 mL/min; spray chamber temperature, 2.7 °C; sample depth and sample introduction flow rate, respectively 5 mm 0.93 mL/min.

Integration times were 0.5 s/point for V, Fe, Se and As, and 0.1 s/point for the other elements.

Data acquisition was possible using the Thermo Scientific QtegraTM Intelligent Scientific Data System software (Thermo Scientific, Waltham, MA, USA). In addition, a seven-point calibration plot with an internal standard normalization was constructed for quantitative analysis. All samples were analysed in triplicate, along with analytical blanks.

### 2.5. DMA-80 Analysis

For the snail samples, the determination of Hg content was carried out with a direct mercury analyser (DMA-80, Milestone S.r.l., Sorisole, Italy).

The DMA-80 analysis was performed in accordance with EPA method 7473 [15]. Precisely, ~100 mg of every sample was initially dried at 250 °C for 3 min and then thermally decomposed at 650 °C for 3 min. Subsequently, having previously constructed a seven-point calibration curve, it was possible to determine the Hg content by working at its typical wavelength, i.e., 253.7.

### 2.6. ICP-MS and DMA-80 Validation Method

In accordance with Eurachem criteria [16], the method was validated in terms of linearity, the limit of detection (LOD), limit of quantification (LOQ), and accuracy, as reported in Table 2.

Linearity was determined by the least-square linear regression method, using multi-standard solutions to construct seven-point calibration curves in the range of 0.5–50.0 μg/L for each individual analyte. For mercury, on the other hand, the concentration range was 1–100 μg/L.

The R^2^ values ranged from 0.9983 (for Na) to 0.9999 (for Cd and Pb). The formulas to calculate LOD and LOQ were as follows: 3.3 σ/b and 10 σ/b, respectively, where σ is the standard deviation of the analytical blank (*n* = 10) and b is the slope of the relative calibration curve. The LOD values ranged from 0.001 mg/Kg to 1.211 mg/Kg; the LOQs from 0.003 mg/kg to 3.996 mg/kg, instead (Table 2). Six replicates were performed on the certified matrix (ERM-CE278k-Mussel Tissue) to determine the accuracy, and the difference between the mean experimental value and the reference value was reported as mean percent recovery. For elements which were not present in the reference material, a known amount of the latter (2.0 mg/Kg) was added.

### 2.7. Statistical Analysis

All mineral data are reported as the mean and standard deviation of three independent determinations. Sample mineral content data are reported as the average of 2 sample data and related standard deviation.

## 3. Results

In this study, 28 mineral elements (Ag, Al, As, B, Ba, Be, Bi, Cd, Co, Cr, Cu, Fe, Hg, K, Li, Na, Mg, Mn, Mo, Ni, Pb, Sb, Se, Sr, Ti, Tl, V, Zn) in both the edible part and the shell of edible land snails commercially available in Southern Italy belonging to *Cernuella virgata*, *Helix aperta* and *Theba pisana* species were evaluated by means of ICP-MS and DMA-80 analysis.

The linearity over the concentration range tested was optimal, exhibiting r^2^ > 0.9982 for all reference standards, as shown in Table 2. Exhibited LOD values ranged from 0.001 to 1.211 mg/kg, while LOQs ranged from 0.003 to 3.996 mg/kg (Table 2), indicating the good sensitivity of the method for sample analysis (Table 2). The accuracy of the analytical procedure, evaluated by using the recovery test, was very good, with percentage recovery values in the 89–101% range. The lowest and the highest average recovery were observed, respectively, for Na (89.67%) and Cd (101.19%) (Table 2).

Quantitative data of the exhibited mineral content are reported in Table 3 and Table 4. Table 3 shows the mineral contents present in the edible parts of the analysed gastropods and Table 4 shows the mineral contents found in the gastropods’ shells. The mineral elements are reported in alphabetical order in both Table 3 and Table 4 and all values are expressed as mg/kg.

Aluminium (Al) contents were very variable, ranging between 2.45 and 2379.78 mg/kg in the shell’s samples, and between 0.01 and 0.83 mg/kg in edible parts.

Arsenic (As) contents were similar in both sample types. Precisely, the content of this element ranged between 0.01 and 0.08 mg/kg in the edible parts, and between 0.01 and 0.21 mg/kg in shells.

Boron (B) was detected in five shell samples of the 12, ranging between 0.10 and 0.86; all edible parts showed a boron level less than the quantification limit instead.

Barium (Ba) concentrations varied among the samples. In fact, the range changed between 0.18 and 28.51 mg/kg in shells, and between 0.18 and 2.61 mg/kg in edible parts.

Beryllium (Be) was detected in only three samples of shells ranging between 0.01 and 0.07 mg/kg. For the other samples, the concentrations of this element were below the LOQ value.

Bismuth (Bi) showed the same levels depending on the type of sample. For the edible part, all gastropods had a concentration of 0.004 mg/kg; shells instead were characterized by values below the quantification limit.

The toxic element cadmium (Cd) was present in small quantities. In the edible part its concentration varied from 0.003 to 0.03 mg/kg, with a sample that showed a level below the LOQ. For the shells, instead, only three samples presented cadmium (0.01–0.03 mg/kg).

Half of the edible part samples contained the same quantity of cobalt (Co), namely 0.003 mg/kg; the other concentrations were below the quantification limit. For the shells, however, the levels ranged between 0.003 and 0.18 mg/kg.

For all samples chromium (Cr) concentrations were obtained: 0.02–0.07 mg/kg for the edible parts; 0.03–0.78 mg/kg for shells.

Higher copper (Cu) and iron (Fe) levels were shown in edible parts than the shells: 22.11–48.20 mg/kg vs. 0.07–3.12 mg/kg for Cu; 253.91–349.73 mg/kg vs. 2.18–508.25 mg/kg.

Mercury (Hg) was quantified only in edible part samples (0.003–0.02 mg/kg).

Potassium (K) was highly present in the edible parts with a concentration range between 783.82–908.44 mg/kg. For the shells, the K levels were important also: 12.37–1483.05 mg/kg.

The lithium (Li) content was similar in both sample types, except in some cases. The ranges were: 0.02–0.06 mg/kg in edible parts; 0.004–0.36 mg/kg in shells.

The magnesium (Mg) level was significantly higher in the edible parts than in the shells. In fact, the concentration range of the former was 352.19–408.33 mg/kg, while that of the latter was 80.73–410.49 mg/kg.

The same trend was shown by the manganese (Mn) content: 20.56–35.72 mg/kg in the edible part; 1.08–36.52 mg/kg in the shells.

Molybdenum (Mo) was below the LOQ in all edible part samples. Instead, half of the shells presented a Mo range between 0.01 and 0.07 mg/kg.

Most edible part samples showed a higher concentration of sodium (Na) than the respective shell. The range, in fact, varied between 553.24 and 626.23 mg/kg in the edible part, and between 37.75 and 601.11 mg/kg in the shells.

Regarding nickel (Ni), the concentration changed in relation to the sample. In the edible part, Ni levels ranged between 0.08 and 0.14 mg/kg. For the shells, instead, two samples presented a nickel content below LOQ value; the range for the others, instead, varied between 0.004 and 2.02 mg/kg.

The lead (Pb) level in the edible part was low (0.003–0.07 mg/kg). A higher content of this element was obtained for some shells that showed a Pb range between 0.01 and 1.42 mg/kg. Only one shell sample presented a lead concentration below the LOQ.

All edible part samples were characterized by a minimum content of antimony (Sb), always lower than the quantification limit. Low concentrations of this element were also obtained for shells, with half of the samples showing a content below LOQ, and the other half an Sb level oscillating between 0.003 and 0.04 mg/kg.

The selenium (Se) content was slightly higher in the edible part of the investigated gastropods, ranging from 0.07 and 0.21 mg/kg. For the shells, however, the Se level varied between 0.01 and 0.02 mg/kg.

Strontium (Sr) levels were abundantly higher in shells samples with a range concentration between 6.34 and 200.13 mg/kg. The meat showed a contribution of Sr between 0.04 and 0.52 mg/kg.

A markedly opposite trend was observed for the titanium (Ti) content, for which the shell samples showed significant concentrations (4.45–419.73 mg/kg) compared to the edible part (n.d.−0.06 mg/kg).

Thallium (Tl) content was low in all samples, many of which had levels below LOQ. The concentration range of this element varied between n.d. and 0.003 mg/kg in the edible part, and between n.d. and 0.02 mg/kg in the shells.

For vanadium (V), the edible part almost always showed very low contents (n.d.−0.003 mg/kg) compared to the shells (n.d.−4.33 mg/kg).

Finally, zinc (Zn) was much more abundant in the edible part (123.68–220.51 mg/kg) than the shells (1.02–3.76 mg/kg).

Different mineral distribution has been observed depending on the species (Table 5). Clearly, the habitat/element uptake relationship is influenced by other factors, such as snail food sources, behaviour, and abiotic factors (e.g., sediment properties) [17]. Relative to the edible part, the most significant differences were obtained for Ba, Cu, K, Mn, Se, Sr e Zn. For barium (Ba), *Theba pisana* and *Cernuella virgata* were characterized by higher levels than *Helix aperta*. Opposite trends were observed for copper (Cu), manganese (Mn) and zinc (Zn) (*Helix aperta* > *Theba pisana* and *Cernuella virgata*). For potassium (K) and selenium (Se), it was *Cernuella virgata* that showed higher levels than the other two species. Finally, for strontium (Sr), the order of abundance was as follows: *Theba pisana* > *Cernuella virgata* > *Helix aperta*. Shifting the focus to the shells, however, highly variable results were obtained among the samples analysed both intra- and interspecies. This again demonstrates the close correlation between the mineral concentration accumulated in the edible part and shell of the different snails with that present in the soil of the area where the snails were collected [2].

## 4. Discussion

The concentration of trace elements was variable among the samples. Higher levels of aluminium were found in the shell, with values varying depending on the sample. Snails of the species *Helix aperta* presented the highest level of this element, followed by *Cernuella virgata* and *Theba pisana*. In the literature, the study conducted by Vukasinovic-Pesic et al. [7] showed a concentration range for aluminium in the shells of three snail species between 445.6 and 512.4 mg/kg. In comparison, only some of the obtained results are comparable. The different availability of Al in the shell samples shows a close correlation with the different soils on which the snails investigated lived, which are responsible for the different intakes of this element in the animal. The Al content, in fact, varies not only according to the type of soil but also to the acidity level of the soil; for example, the level of this element increases as the pH decreases [18]. Furthermore, from the data obtained, it could be assumed that snail samples tend to accumulate aluminium in their shells, given the low Al content in the edible parts.

A similar argument can be made for barium (Ba). This element, in fact, showed significantly higher concentrations in the shells rather than in the edible part. In this case, the high presence of this element can be related to two plausible explanations: absorption from the soil and the possible use of plant protection chemicals based on Ba compounds such as hydroxide, chloride, or carbonate [18] in the origin area of the analysed species.

The cadmium (Cd) content was very low in all samples analysed. EU Commission Regulation 2021/1323 [19] sets a maximum limit for Cd in bivalve molluscs of 1.0 mg/kg fresh weight. Our results were well within this value. Cadmium levels were also lower than in other studies [5,17,20].

A further clear difference in content was observed for copper (Cu). For this element, the highest levels were obtained for snail meat samples. The results for the edible part of the snail were comparable with those reported by Engmann et al. [21] (mean Cu content: 33 mg/kg), Mahmutovic et al. [20] (Cu: 9.74–26.62 mg/kg), Nkansah et al. [3] (Cu: 7.3–38.3 mg/kg), but significantly lower than the study by Proum et al. [17] (mean Cu: 217.68 mg/kg). Furthermore, all studies reinforced the hypothesis of this study, i.e., the presence of a lower copper content in shells than in meat. The greatest accumulation of this element in meat is due to the incorporation of copper into proteins, including respiratory proteins and enzymes [17,22,23].

Most meat samples showed a higher iron (Fe) content than the corresponding shells. In the latter, the iron content was quite variable, depending on the snail’s gender and geographical origin. This influence was also observed in another study, conducted by Proum et al. [17], where the iron content in the shells varied between 5 and 1000 mg/kg. In relation to the edible part, our Fe contents were almost always far higher than in two studies: that of Nkansah et al. [3], with a range of Fe concentrations between 5.75–26.64 mg/100 g, and that conducted by Zarai et al. [24], which indicated an average iron content of 81.00 mg/kg. The results were, however, comparable to those obtained by Proum et al. [17]. Once again, this variability demonstrates the close connection with the type of snail, the geographical origin, and the habitat in which the analysed species grows. Furthermore, the data obtained indicate that the meat of our snails is an excellent source of iron in the diet.

Regarding mercury (Hg), there is no limit for this element in land snails. There is, however, EU Commission Regulation 2022/617 [25], which indicates a maximum mercury content in marine gastropods of 0.30 mg/kg. Given the low contribution of Hg found in the analysed samples, the limit was perfectly respected. The mercury contribution was also shown to be low by comparison with the literature [20].

As for the content of macro-elements (K, Na and Mg), however, for most samples, the highest concentrations were observed in the edible part. This finding mirrors the trend reported by other studies in the literature, as shown, for example, by the study conducted by Nkansah et al. on the determination of minerals and the proximal composition of the meat and shells of three snail species [3]. Furthermore, our levels of K, Na and Mg were significantly lower than in two studies in the literature. The first one, conducted by Tonfack Djikeng et al. [26], reported average potassium, sodium, and magnesium contents of 280.40 mg/100 g, 121.20 mg/100 g and 117.20 mg/100 g, respectively, in meat samples of snails of the genus *Archachatina marginata*. The second, performed by Ab Lah et al. [27], indicated levels of K (2.73–3.33 mg/g), Na (2.70–4.00 mg/g) and Mg (0.65–0.77 mg/g) in three different species of turban snail. This shows how the macro-nutrient content varies not only according to the snail species analysed, but also in relation to the habitat in which they live. Although the data obtained from the analysis of our samples were lower than those in the literature, the snails in this study were found to be a good source of macro-nutrients (K, Na and Mg).

A similar argument can be made for the manganese (Mn) contents in almost all the snail meat samples analysed, which were significantly higher than in the shells. This shows the higher accumulation of this element in the edible part of the snail. The abundance of Mn is closely related to the type of soil on which the snails live. Indeed, acidic soils contain a high percentage of Mn^2+^ ions and may facilitate its absorption. This close correlation with the soil confirms the possibility of using these species as indicators of the degree of environmental pollution. However, the behaviour observed in our study was not the same as that reported by Proum et al. [17] who reported almost similar contents of the two parts (an average of 97.77 mg/kg in the flesh vs. 90.34 mg/kg in the shell).

Lead (Pb) also had variable and higher contents in the shells rather than in the edible parts. Some shell samples had Pb concentrations even above 1 mg/kg. These results proved to be far lower than those reported by Proum et al. (average Pb content: 181.29 mg/kg) [17]. Regarding the edible part, the concentrations were very low. For this element, Regulation 1881/2006 of the European Commission [28] and subsequent amendments set maximum limits for various food groups. Although no specific regulatory limit is specified for the category “land snails”, reference can be made to the group “bivalve molluscs”. Currently, for this category, EU Regulation 2021/1317 [29] sets a maximum lead content of 1.50 mg/kg of fresh product. Therefore, our results for snail meat fall within this threshold.

The same trend was also observed for strontium (Sr). This element, in fact, was more accumulated in shell samples. Probably, this accumulation is due to the strontium presence in the soil where the snails lived. The important content of this element is agreed with the study of Caetano et al. [5], which analysed the nutritional and toxicity profiles of two species of land snail, *Theba pisana* and *Otala lacteal*, from Morocco.

Shell samples are characterized by a higher level of titanium (Ti) than meat. There are no studies in the literature that have addressed the determination of this element. However, a possible explanation for the high Ti content in the shells could be related to the important input from the soil.

The vanadium (V) content was very low in the edible part and often below the LOQ value. A different trend was observed, however, in the shells, for which the content of this element was variable. It was below the limit of quantification in three of the twelve samples, while for the others, the concentration ranges between 0.03 and 4.33 mg/kg. Some of the results obtained for the snail shells investigated agreed with Al-Saad et al. [30], who evaluated toxic elements in the fresh part and shells of *Bellamya bengalensis* snails from the Shatt Al-Arab River area in Iraq, reporting V contents between 1.80 and 4.85 mg/kg.

Regarding zinc content, the highest levels were obtained for the edible parts. This agreed with what was reported by Nkansah et al. [3]. Our study also showed that Zn was present in significant quantities in all samples of the edible part of the species analysed, with sample 10 of the genus *Helix aperta*, purchased in Messina but of Tunisian origin, having the highest concentration (220.51 ± 2.83 mg/kg). Being purely nocturnal species, the high concentration of zinc obtained in the meat of the snail samples analysed could be explained by their need to store significant amounts of Zn, useful for life adaptation in dark conditions [21]. Furthermore, the results obtained for all meat samples were in some cases superior to those of other studies in the literature on species of different genera and geographical origin [3,17,31], but comparable to other investigations [21]. This shows how these two factors are fundamental and influence the different mineral content of these animals.

For the remaining elements (Ag, As, B, Be, Bi, Co, Cr, Li, Mo, Ni, Sb, Se, Tl), the levels were low and comparable with those in the literature [5,20].

## 5. Conclusions

This study aimed at monitoring the levels of trace elements in land edible snails commercially available in Southern Italian markets and commonly used in local food recipes. Concerning the contents of toxic and potentially toxic elements and the susceptibility of edible snails with respect to environmental contamination and terrestrial biota, constant monitoring and further investigation should be taken into consideration to minimize risks to human health.

## Figures and Tables

**Table 1:** Samples investigated and related purchasing and origin areas.

Sample *n*.	*Species*	Purchasing Area	Origin Area
1	*Theba pisana*	Palermo	n.d. ^1^
2	*Theba pisana*	Palermo	n.d. ^1^
3	*Helix aperta*	Messina	Tunisia
4	*Helix aperta*	Messina	Tunisia
5	*Helix aperta*	Messina	Tunisia
6	*Helix aperta*	Messina	Tunisia
7	*Helix aperta*	Messina	Tunisia
8	*Helix aperta*	Messina	Tunisia
9	*Helix aperta*	Messina	Tunisia
10	*Helix aperta*	Messina	Tunisia
11	*Cernuella virgata*	Palermo	n.d. ^1^
12	*Cernuella virgata*	Palermo	n.d. ^1^

^1^ n.d.: not declared.

**Table 2:** Analytical parameters for method validation.

Element	R^2^	LOD (mg/kg)	LOQ (mg/kg)	ERM-CE278k-Mussel Tissue		
				Experimental Value (mg/kg)	Expected Value (mg/kg)	Recovery (%)
Ag	0.9995	0.001	0.003	0.044	0.040	90.91
Al *	0.9996	0.002	0.007	2.00	1.97	98.50
As	0.9998	0.001	0.003	6.7	6.6	98.51
B *	0.9996	0.001	0.003	2.00	1.95	97.50
Ba *	0.9995	0.003	0.010	2.00	1.98	99.00
Be *	0.9995	0.001	0.003	2.00	1.95	97.50
Bi *	0.9996	0.001	0.003	2.00	1.96	98.00
Cd	0.9999	0.001	0.003	0.336	0.340	101.19
Co	0.9997	0.001	0.003	0.21	0.20	95.24
Cr	0.9996	0.004	0.013	0.73	0.71	97.26
Cu *	0.9991	0.012	0.040	2.00	1.98	99.00
Fe	0.9995	0.015	0.050	161	155	96.27
Hg	0.9997	0.001	0.003	0.071	0.067	94.37
K	0.9985	0.304	1.003	5370	4869	90.67
Li *	0.9996	0.001	0.003	2.00	1.95	97.50
Mg	0.9990	0.043	0.142	1510	1428	94.57
Mn	0.9997	0.010	0.033	4.88	4.79	98.16
Mo *	0.9998	0.002	0.007	2.00	1.97	98.50
Na	0.9983	1.211	3.996	13,900	12,464	89.67
Ni	0.9996	0.001	0.003	0.69	0.66	95.65
Pb	0.9999	0.001	0.003	2.18	2.20	100.92
Sb *	0.9994	0.001	0.003	2.00	1.96	98.00
Se	0.9992	0.011	0.036	1.62	1.58	97.53
Sr	0.9994	0.009	0.030	19.00	17.77	93.53
Ti *	0.9993	0.002	0.007	2.00	1.99	99.50
Tl *	0.9998	0.001	0.003	2.00	1.96	98.00
V *	0.9995	0.001	0.003	2.00	1.95	97.50
Zn	0.9991	0.050	0.165	71.00	67.34	94.85

* Not present in the certified matrix. Added later to the matrix.

**Table 3:** Mineral contents in edible parts of gastropod samples, expressed as mg/kg.

Mineral (mg/kg)	1	2	3	4	5	6	7	8	9	10	11	12
	*T. p*	*T. p*	*H. a*	*H. a*	*H. a*	*H. a*	*H. a*	*H. a*	*H. a*	*H. a*	*C. v*	*C. v*
**Ag**	n/d	n/d	n/d	n/d	n/d	0.01 *	n/d	n/d	n/d	n/d	n/d	n/d
**Al**	0.01 *	0.01 *	0.40 ± 0.07	0.05 ± 0.01	0.48 ± 0.12	0.04 ± 0.01	0.05 ± 0.01	0.04 ± 0.01	0.13 ± 0.03	0.83 ± 0.15	0.06 ± 0.01	0.18 ± 0.05
**As**	0.02 *	0.02 ± 0.01	0.01 *	0.03 ± 0.02	0.03 ± 0.01	0.03 ± 0.01	0.08 ± 0.02	0.07 ± 0.02	0.06 ± 0.01	0.06 ± 0.02	0.03 ± 0.02	0.03 ± 0.01
**B**	n/d	n/d	n/d	n/d	n/d	n/d	n/d	n/d	n/d	n/d	n/d	n/d
**Ba**	0.89 ± 0.07	0.86 ± 0.12	0.44 ± 0.08	0.35 ± 0.08	0.18 ± 0.03	0.26 ± 0.09	1.18 ± 0.29	0.50 ± 0.10	1.42 ± 0.40	0.96 ± 0.19	1.39 ± 0.29	2.61 ± 0.45
**Be**	n/d	n/d	n/d	n/d	n/d	n/d	n/d	n/d	n/d	n/d	n/d	n/d
**Bi**	0.004 *	0.004 *	0.004 *	0.004 *	0.004 *	0.004 *	0.004 *	0.004 *	0.004 *	0.004 *	0.004 *	0.004 *
**Cd**	0.004 *	0.003 *	n/d	0.004 *	0.01 *	0.01 *	0.03 ± 0.01	0.03 ± 0.01	0.01 *	0.03 ± 0.01	0.01 *	0.01 *
**Co**	n/d	n/d	n/d	n/d	0.003 *	n/d	0.003 *	0.003 *	0.003 *	n/d	0.003 *	0.003 *
**Cr**	0.02 ± 0.02	0.03 ± 0.01	0.03 ± 0.01	0.04 *	0.05 ± 0.01	0.05 ± 0.01	0.07 ± 0.01	0.07 *	0.07 ± 0.01	0.07 ± 0.01	0.04 ± 0.01	0.04 *
**Cu**	24.84 ± 0.59	22.11 ± 0.43	23.13 ± 0.30	36.55 ± 0.79	34.23 ± 0.66	33.18 ± 0.71	42.23 ± 1.05	46.15 ± 1.71	46.55 ± 1.06	48.20 ± 2.70	29.34 ± 2.10	26.23 ± 1.59
**Fe**	253.91 ± 0.70	268.11 ± 0.97	270.82 ± 1.17	290.62 ± 1.34	294.39 ± 1.61	292.11 ± 1.61	326.17 ± 2.16	349.73 ± 1.93	342.71 ± 1.21	346.44 ± 1.47	304.11 ± 1.99	307.84 ± 2.37
**Hg**	0.004 *	0.004 *	0.003 *	0.003 *	0.003 *	0.004 *	0.004 *	0.004 *	0.003 *	0.004 *	0.01 *	0.02 ± 0.01
**K**	786.23 ± 1.92	783.82 ± 2.22	804.25 ± 1.83	803.64 ± 2.59	801.61 ± 3.38	858.42 ± 4.61	866.09 ± 3.69	867.09 ± 2.99	861.15 ± 2.78	876.71 ± 3.30	908.44 ± 3.67	907.85 ± 4.05
**Li**	0.02 ± 0.01	0.03 ± 0.01	0.03 ± 0.01	0.04 ± 0.01	0.04 ± 0.01	0.04 ± 0.01	0.05 ± 0.01	0.05 ± 0.01	0.06 ± 0.01	0.06 ± 0.01	0.03 ± 0.01	0.03 ± 0.01
**Mg**	352.76 ± 0.74	352.19 ± 1.10	373.85 ± 1.16	364.46 ± 1.51	363.76 ± 2.19	402.16 ± 2.62	393.68 ± 1.48	404.63 ± 3.55	392.99 ± 2.23	408.33 ± 2.33	368.80 ± 3.38	365.23 ± 2.11
**Mn**	20.56 ± 0.30	23.65 ± 0.29	24.53 ± 0.38	27.09 ± 0.43	28.67 ± 0.30	27.53 ± 0.48	32.16 ± 0.80	31.92 ± 0.82	35.72 ± 0.81	33.72 ± 0.61	26.56 ± 0.79	25.10 ± 0.38
**Mo**	n/d	n/d	n/d	n/d	n/d	n/d	n/d	n/d	n/d	n/d	n/d	n/d
**Na**	564.63 ± 1.62	563.07 ± 1.17	584.09 ± 1.51	583.36 ± 2.28	589.42 ± 1.86	612.15 ± 2.16	622.29 ± 1.83	622.27 ± 2.22	626.23 ± 1.67	616.43 ± 2.33	554.16 ± 1.95	553.24 ± 1.96
**Ni**	0.09 ± 0.01	0.09 ± 0.01	0.08 ± 0.01	0.11 ± 0.02	0.11 ± 0.02	0.11 ± 0.02	0.14 ± 0.04	0.14 ± 0.03	0.14 ± 0.03	0.14 ± 0.04	0.10 ± 0.02	0.10 ± 0.02
**Pb**	0.003 *	0.003 *	0.003 *	0.01 *	0.01 *	0.003 *	0.07 ± 0.01	0.02 ± 0.01	0.02 ± 0.01	0.01 *	0.003 *	0.003 *
**Sb**	n/d	n/d	n/d	n/d	n/d	n/d	n/d	n/d	n/d	n/d	n/d	n/d
**Se**	0.07 ± 0.01	0.07 ± 0.01	0.07 ± 0.01	0.11 ± 0.02	0.11 ± 0.03	0.12 ± 0.04	0.14 ± 0.04	0.15 ± 0.04	0.16 ± 0.04	0.15 ± 0.03	0.21 ± 0.05	0.20 ± 0.04
**Sr**	0.52 ± 0.09	0.38 ± 0.03	0.16 ± 0.03	0.04 ± 0.01	0.13 ± 0.02	0.26 ± 0.04	0.30 ± 0.06	0.13 ± 0.03	0.19 ± 0.04	0.22 ± 0.04	0.06 ± 0.01	0.10 ± 0.02
**Ti**	0.06 ± 0.01	0.06 ± 0.01	0.02 *	n/d	0.04 ± 0.01	0.02 *	0.02 *	0.01 *	0.04 ± 0.01	0.02 ± 0.01	0.03 *	0.02 *
**Tl**	0.003 *	0.003 *	n/d	n/d	n/d	n/d	n/d	0.003 *	0.003 *	n/d	0.003 *	n/d
**V**	n/d	n/d	0.003 *	0.003 *	0.003 *	0.003 *	0.003 *	0.003 *	0.003 *	0.003 *	n/d	n/d
**Zn**	129.88 ± 1.05	123.68 ± 0.90	124.56 ± 1.07	141.13 ± 0.68	146.80 ± 0.95	150.21 ± 2.45	203.45 ± 2.07	208.95 ± 1.74	217.05 ± 1.99	220.51 ± 2.83	187.72 ± 1.85	184.33 ± 1.80

*T. p*: *Theba pisana*; *H. a*: *Helix aperta*; *C. v*: *Cernuella virgata*. n/d = not detected (<LOQ); * = SD < 0.01.

**Table 4:** Mineral contents in the gastropod shells samples expressed as mg/kg.

Mineral (mg/kg)	1	2	3	4	5	6	7	8	9	10	11	12
	*T. p*	*T. p*	*H. a*	*H. a*	*H. a*	*H. a*	*H. a*	*H. a*	*H. a*	*H. a*	*C. v*	*C. v*
**Ag**	n/d	n/d	n/d	n/d	n/d	n/d	0.02 *	0.01 *	n/d	0.01 *	n/d	0.01 *
**Al**	2.45 ± 0.36	3.76 ± 0.35	73.44 ± 0.35	28.97 ± 0.33	4.75 ± 0.22	149.61 ± 0.40	531.65 ± 0.68	2379.78 ± 1.97	164.07 ± 0.40	783.32 ± 0.46	174.70 ± 0.69	125.08 ± 0.56
**As**	0.01 *	0.02 ± 0.01	0.03 ± 0.01	0.03 ± 0.01	0.02 ± 0.01	0.04 ± 0.01	0.08 ± 0.02	0.11 ± 0.04	0.05 ± 0.01	0.21 ± 0.04	0.04 ± 0.01	0.03 ± 0.01
**B**	n/d	n/d	n/d	n/d	n/d	0.17 ± 0.04	0.86 ± 0.04	0.64 ± 0.06	n/d	0.28 ± 0.04	0.10 ± 0.02	0.32 ± 0.04
**Ba**	2.07 ± 0.08	3.21 ± 0.11	2.11 ± 0.06	0.90 ± 0.09	0.18 ± 0.05	3.86 ± 0.14	12.00 ± 0.34	8.97 ± 0.15	10.86 ± 0.27	28.51 ± 0.49	3.86 ± 0.17	6.83 ± 0.29
**Be**	n/d	n/d	n/d	n/d	n/d	n/d	0.07 ± 0.01	0.02 ± 0.01	n/d	0.01 *	n/d	n/d
**Bi**	n/d	n/d	n/d	n/d	n/d	n/d	n/d	n/d	n/d	n/d	n/d	n/d
**Cd**	n/d	n/d	n/d	n/d	n/d	n/d	0.01 *	n/d	n/d	0.03 ± 0.01	n/d	0.02 *
**Co**	0.003 *	0.003 *	0.02 *	0.01 *	n/d	0.04 *	0.11 *	0.16 ± 0.01	0.04 ± 0.01	0.18 ± 0.01	0.07 ± 0.01	0.07 ± 0.01
**Cr**	0.03 ± 0.01	0.03 ± 0.01	0.14 ± 0.02	0.11 ± 0.02	0.12 ± 0.03	0.51 ± 0.04	0.58 ± 0.06	0.47 ± 0.05	0.78 ± 0.04	0.38 ± 0.07	0.19 ± 0.06	0.23 ± 0.05
**Cu**	1.39 ± 0.32	1.45 ± 0.36	2.90 ± 0.43	3.08 ± 0.13	3.12 ± 0.22	0.71 ± 0.15	1.33 ± 0.22	1.78 ± 0.26	0.30 ± 0.09	1.83 ± 0.15	0.26 ± 0.02	0.07 ± 0.01
**Fe**	3.88 ± 0.38	2.86 ± 0.21	43.71 ± 0.66	21.40 ± 0.55	2.18 ± 0.19	87.93 ± 1.01	379.46 ± 1.15	401.66 ± 0.76	107.41 ± 1.02	508.25 ± 1.06	103.03 ± 0.59	31.36 ± 0.40
**Hg**	n/d	n/d	n/d	n/d	n/d	n/d	n/d	n/d	n/d	n/d	n/d	n/d
**K**	66.38 ± 0.24	50.42 ± 0.52	22.74 ± 0.32	12.37 ± 0.20	23.51 ± 0.33	243.92 ± 0.39	1396.47 ± 2.12	1482.20 ± 1.85	128.58 ± 0.48	341.37 ± 0.44	1483.05 ± 0.76	1366.09 ± 0.61
**Li**	0.004 *	0.01 *	0.02 ± 0.01	0.02 ± 0.01	0.02 ± 0.01	0.06 ± 0.01	0.20 ± 0.03	0.36 ± 0.04	0.06 ± 0.03	0.30 ± 0.04	0.03 ± 0.01	0.02 ± 0.01
**Mg**	108.61 ± 0.41	99.56 ± 0.22	93.90 ± 0.42	82.69 ± 0.53	108.28 ± 0.27	80.83 ± 0.39	309.19 ± 0.83	410.49 ± 1.00	80.73 ± 0.71	111.75 ± 0.60	94.44 ± 0.60	105.71 ± 0.48
**Mn**	1.67 ± 0.16	1.47 ± 0.18	1.60 ± 0.15	1.77 ± 0.28	1.08 ± 0.14	3.65 ± 0.19	8.76 ± 0.27	36.52 ± 0.32	6.48 ± 0.14	28.98 ± 1.44	4.56 ± 0.27	4.67 ± 0.30
**Mo**	n/d	n/d	n/d	n/d	n/d	0.01 *	0.04 *	0.05 ± 0.01	0.06 ± 0.02	0.07 ± 0.02	n/d	0.01 *
**Na**	46.80 ± 0.82	37.75 ± 0.53	49.17 ± 0.56	69.12 ± 0.38	92.68 ± 0.32	143.83 ± 0.60	433.65 ± 0.47	601.11 ± 0.42	43.66 ± 0.59	126.52 ± 0.32	308.60 ± 0.47	497.96 ± 0.65
**Ni**	0.004 *	0.01 *	0.08 ± 0.01	n/d	n/d	0.11 ± 0.01	1.09 ± 0.02	1.25 ± 0.02	0.55 ± 0.01	2.02 ± 0.02	0.31 ± 0.02	0.09 ± 0.01
**Pb**	0.02 ± 0.01	0.01 *	0.05 ± 0.02	0.02 ± 0.01	0.01 *	0.03 ± 0.01	0.12 ± 0.03	0.19 ± 0.03	0.20 ± 0.03	1.42 ± 0.04	0.02 ± 0.01	n/d
**Sb**	n/d	n/d	0.003 *	n/d	n/d	0.003 *	0.01 *	0.01 *	0.01 *	0.04 ± 0.01	n/d	n/d
**Se**	0.01 *	0.01 *	0.01 *	0.02 ± 0.01	0.01 *	0.01 *	0.02 *	0.02 ± 0.01	0.02 *	0.02 ± 0.01	0.01 *	0.01 *
**Sr**	72.35 ± 0.39	82.96 ± 0.49	22.74 ± 0.44	13.53 ± 0.36	6.34 ± 0.51	139.35 ± 0.39	165.38 ± 0.64	200.13 ± 0.49	84.05 ± 0.37	158.29 ± 0.61	85.72 ± 0.50	50.08 ± 0.57
**Ti**	21.45 ± 0.60	32.09 ± 0.36	19.70 ± 0.42	11.82 ± 0.11	4.45 ± 0.13	86.63 ± 0.45	313.68 ± 0.79	419.59 ± 1.55	59.31 ± 0.66	106.80 ± 0.60	419.73 ± 0.61	263.24 ± 0.57
**Tl**	n/d	n/d	n/d	n/d	n/d	n/d	0.004 *	0.01 *	0.01 *	0.02 ± 0.01	n/d	n/d
**V**	n/d	n/d	0.15 ± 0.04	0.03 *	n/d	0.48 ± 0.11	4.33 ± 0.35	3.81 ± 0.41	0.50 ± 0.22	3.61 ± 0.34	0.36 ± 0.14	0.20 ± 0.06
**Zn**	1.02 ± 0.12	1.06 ± 0.05	2.03 ± 0.09	2.01 ± 0.10	2.02 ± 0.10	3.02 ± 0.14	3.66 ± 0.13	3.76 ± 0.13	3.15 ± 0.17	3.50 ± 0.21	2.02 ± 0.15	2.36 ± 0.18

*T. p*: *Theba pisana*; *H. a*: *Helix aperta*; *C. v*: *Cernuella virgata*. n/d = not detected (<LOQ); * = SD < 0.01. Silver (Ag) content was detected in four samples of shells with low contents falling within 0.02 and 0.01 mg/kg. Only one sample of edible parts showed a quantifiable silver level (0.01 mg/kg).

**Table 5:** Average mineral contents in different species of gastropods, expressed as mg/kg.

Mineral (mg/kg)	*Theba pisana*		*Helix aperta*		*Cernuella virgata*	
	Shell	Meat	Shell	Meat	Shell	Meat
**Ag**	n/d	n/d	0.01 *	n/d	0.01 *	n/d
**Al**	3.11 ± 0.36	0.01 *	514.45 ± 0.60	0.25 ± 0.05	149.89 ± 0.63	0.12 ± 0.03
**As**	0.02 ± 0.01	0.02 ± 0.01	0.07 ± 0.02	0.05 ± 0.02	0.04 ± 0.01	0.03 ± 0.02
**B**	n/d	n/d	0.49 ± 0.05	n/d	0.21 ± 0.03	n/d
**Ba**	2.64 ± 0.10	0.88 ± 0.09	8.16 ± 0.20	0.66 ± 0.16	5.35 ± 0.23	2.00 ± 0.37
**Be**	n/d	n/d	0.03 ± 0.01	n/d	n/d	n/d
**Bi**	n/d	0.004 *	n/d	0.004 *	n/d	0.004 *
**Cd**	n/d	0.004 *	0.02 ± 0.01	0.02 ± 0.01	0.01 *	0.01 *
**Co**	0.003 *	n/d	0.08 ± 0.01	0.003 *	0.07 ± 0.01	0.003 *
**Cr**	0.03 ± 0.01	0.03 ± 0.02	0.39 ± 0.04	0.06 ± 0.01	0.21 ± 0.06	0.04 ± 0.01
**Cu**	1.42 ± 0.34	23.48 ± 0.51	1.88 ± 0.21	38.78 ± 1.12	0.17 ± 0.02	27.79 ± 1.85
**Fe**	3.37 ± 0.30	261.01 ± 0.84	194.00 ± 0.80	314.12 ± 1.68	67.20 ± 0.50	305.98 ± 2.18
**Hg**	n/d	0.004 *	n/d	0.004 *	n/d	0.02 ± 0.01
**K**	58.40 ± 0.38	785.03 ± 2.07	456.40 ± 0.77	842.37 ± 3.15	1424.57 ± 0.69	908,15 ± 3.86
**Li**	0.01 *	0.03 ± 0.01	0.13 ± 0.02	0.05 ± 0.01	0.03 ± 0.01	0.03 ± 0.01
**Mg**	104.09 ± 0.32	352.48 ± 0.92	159.73 ± 0.59	387.98 ± 2.13	100.08 ± 0.54	367.02 ± 2.75
**Mn**	1.57 ± 0.17	22.11 ± 0.30	11.11 ± 0.37	30.17 ± 0.58	4.62 ± 0.29	25.83 ± 0.59
**Mo**	n/d	n/d	0.05 ± 0.02	n/d	0.01 *	n/d
**Na**	42.28 ± 0.68	563.85 ± 1.40	194.97 ± 0.46	607.03 ± 1.98	403.28 ± 0.56	553.70 ± 1.96
**Ni**	0.01 *	0.09 ± 0.01	0.85 ± 0.02	0.12 ± 0.03	0.20 ± 0.02	0.10 ± 0.02
**Pb**	0.02 ± 0.01	0.003 *	0.26 ± 0.03	0.02 ± 0.01	0.02 ± 0.01	0.003 *
**Sb**	n/d	n/d	0.01 *	n/d	n/d	n/d
**Se**	0.01 *	0.07 ± 0.01	0.02 ± 0.01	0.13 ± 0.03	0.01 *	0.21 ± 0.05
**Sr**	77.66 ± 0.44	0.45 ± 0.06	95.88 ± 0.48	0.18 ± 0.03	67.90 ± 0.54	0.08 ± 0.02
**Ti**	26.77 ± 0.48	0.06 ± 0.01	127.75 ± 0.59	0.02 ± 0.01	341.49 ± 0.59	0.03 *
**Tl**	n/d	0.003 *	0.01 *	0.003 *	n/d	0.003 *
**V**	n/d	n/d	1.84 ± 0.25	0.003 *	0.28 ± 0.10	n/d
**Zn**	1.04 ± 0.09	126.78 ± 0.98	2.89 ± 0.13	176.58 ± 1.72	2.19 ± 0.17	186.03 ± 1.83

n/d = not detected (<LOQ); * = SD < 0.01.

## Data Availability

Not applicable.
